# Multi-Targeting Andrographolide, a Novel NF-κB Inhibitor, as a Potential Therapeutic Agent for Stroke

**DOI:** 10.3390/ijms18081638

**Published:** 2017-07-27

**Authors:** Chih-Hao Yang, Ting-Lin Yen, Chia-Yuan Hsu, Philip-Aloysius Thomas, Joen-Rong Sheu, Thanasekaran Jayakumar

**Affiliations:** 1 Department of Pharmacology, Taipei Medical University, Taipei 110, Taiwan; chyang@tmu.edu.tw (C.-H.Y.); d119096015@tmu.edu.tw (T.-L.Y.); 2 Division of Cardiology, Department of Internal Medicine, Cathay General Hospital, Taipei 200, Taiwan; 3 Department of Life Science, College of Life Sciences, National Chung Hsing University, Taichung 402, Taiwan; gordanmilke1003@gmail.com; 4 Department of Ocular Microbiology, Institute of Ophthalmology, Joseph Eye Hospital, Tiruchirappalli 620001, Tamil Nadu, India; thomasdiagnosticcentre@gmail.com; 5 Graduate Institute of Clinical Medicine, Taipei Medical University, Taipei 110, Taiwan

**Keywords:** andrographolide, neuroprotection, stroke, multi-targets, signaling pathways

## Abstract

A key focus in the field of drug discovery has been motivated by the neuroprotection of natural compounds. Cerebral ischemia is a multifaceted pathological process with a series of mechanisms, and a perspective for the development of neuroprotectants from traditional herbal medicine or natural products is a promising treatment for this disease. Natural compounds with the effects of anti-oxidation, anti-inflammation, anti-apoptosis, and neurofunctional regulation exhibit therapeutic effects on experimental ischemic brain injury. Conferring to the pharmacological mechanisms underlying neuroprotection, a study found that androgapholide, a diterpene lactone compound, exhibits varying degrees of neuroprotective activities in both in vitro and in vivo experimental models of stroke. The neuroprotective mechanisms of andrographolide are suggested as: (I) increasing nuclear factor E2-related factor 2-heme oxygenase (Nrf2-HO-1) expression through p38-mitogen activated protein kinase (MAPK) regulation, (II) inducing cerebral endothelial cells (CEC) apoptosis and caspase-3 activation, (III) down regulating Bax, inducible nitric oxide synthase (iNOS), and (IV) inhibiting hydroxyl radical (OH^−^) formation, and activating transcription factor NF-κB signaling pathways. Recently, several researchers have also been trying to unveil the principal mechanisms involved in the neuroprotective effects of andrographolide. Therefore, this review aims to summarize an overview on the neuroprotective effects of andrographolide and exemplifies the essential mechanisms involved. This paper can provide information that andrographolide drug discovery may be a promising strategy for the development of a novel class of neuroprotective drug.

## 1. Introduction

Ischemic brain injury, one of the third leading causes of death and the primary cause of adult disability worldwide [1], has attracted increased attention in the field of drug discovery. Cerebral ischemia is frequently initiated by transient or permanent decline of cerebral blood flow created by thrombotic or thromboembolic arterial occlusions. Thrombolytic therapy proposed to repair cerebral perfusion is considered the main rational therapeutic strategy for ischemic brain injury [1]. Nevertheless, reperfusion after thrombolytic therapy frequently leads to a series of cellular, biochemical and metabolic consequences of cerebral ischemia, including intracellular reactive oxygen species (ROS) generation, calcium overload, excitotoxicity in cell injury and inflammation, which ultimately leads to apoptosis irreversible brain injury. Many neuroprotective agents are designed to protect the brain from injury after ischemia-reperfusion or to delay the pathological process [2,3].

Recent studies have encouraged investigation of the possible efficiency of natural compounds to prevent neurological disorders. Some beneficial phytochemicals, especially polyphenols such as quercetin, catechin and resveratrol, display protective abilities in various animal models of neurological disorders. The principal mechanisms include ischemic preconditioning, antioxidation, anti-inflammation, inhibition of microglia recruitment; in terms of underlying mechanisms, the preconditioning or neurohormone pathways seem most attractive. Examples of such preconditioning or neurohormone pathways include those involving cell-survival signaling kinases, the transcription factor NF-E2-related factor 2 (Nrf2) and cAMP-response element binding protein (CREB), as well as histone deacetylases of the sirtuin family. Activation of these pathways protect neurons against injury by upregulating the antioxidant defense, neurotrophic factors and protein chaperones which can support cells to withstand stress [4,5,6]. Many effective components from traditional herbs have been demonstrated to show neuroprotection against ischemic brain injury in experimental studies. According to the pharmacological mechanisms elucidated in numerous reports, studies have evaluated the natural product andrographolide that possess protective effects on ischemic stroke and characterized promising targets for this disease.

### 1.1. Androrapholide in Neuroprotection

**Figure 1:**
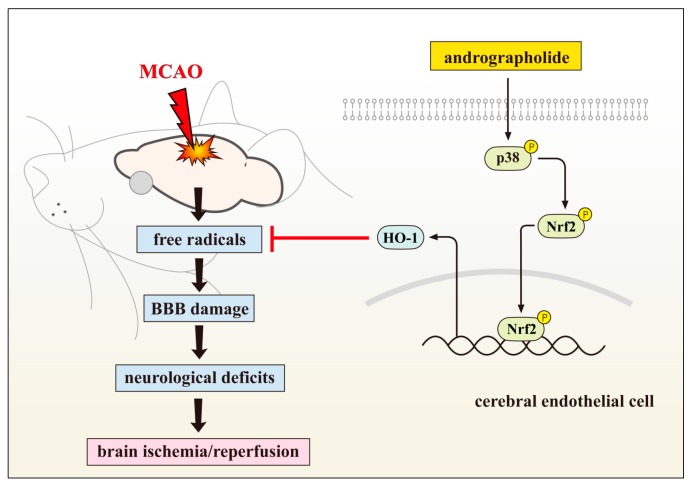
Andrographolide expressively suppressed free radical formation, blood-brain barrier (BBB) disruption, and brain infarction in middle cerebral artery occlusion (MCAO)-insulted rats. The mechanism of this effect was attributable to HO-1 activation. From this study, it is established that andrographolide provides protection against MCAO-induced brain ischemic injury via increased Nrf2-HO-1 expression through p38MAPK regulation.

*Andrographis paniculata* (*A. paniculata*) is a traditional Chinese medicine and has immunological, antibacterial, antiviral, anti-inflammatory, antithrombotic, and hepato protective properties [7,8]. Andrographolide (C_20_H_30_O_5_) is the primary active component of *A. paniculata*, which is widely used in China and other parts of Asia for the treatment of upper respiratory tract infections due to its potent anti-inflammatory activity [9]. Previous study showed that andrographolide reduced infarct volume in the stroke model of rat through the suppression of NF-κB and microglial activation and reduced the production of cytokines and pro-inflammatory factors [10]. In that study, andrographolide (0.1 mg/kg), treated intraperitoneally (i.p) 1 h after permanent middle cerebral artery occlusion (pMCAO), reduced infarct volume with a maximum reduction of approximately 50%. Andrographolide also reduced neurological deficits score by reflecting a correlation between infarct volume and neurological deficits. pMCAO was found to induce activation of microglia and elevate tumour necrosis factor (TNF)-α, interleukin (IL)-1β and prostaglandin (PG) E2 in the ischaemic brain areas. Andrographolide (0.1 mg/kg) was found to be significantly attenuated these effects [10]. In addition, this compound has been shown to reduce inflammation-mediated dopaminergic neurodegeneration in mesencephalic neuron-glia cultures by inhibiting microglial activation, this result implies that andrographolide may have clinical use in treating Parkinson’s disease [11]. The recent study showed that andrographolide at 10 μM increased HO-1 protein and messenger RNA expressions, Nrf2 phosphorylation, and its nuclear translocation in cerebral endothelial cells (CECs), and these activities were found to be disrupted by a p38MAPK inhibitor, SB203580, but not by the extracellular signal-regulated kinase (ERK1/2) inhibitor PD98059 or c-Jun amino-terminal kinase (JNK) inhibitor SP600125 [12]. This study also found that andrographolide expressively suppressed free radical formation, blood-brain barrier (BBB) disruption, and brain infarction in middle cerebral artery occlusion (MCAO)-insulted rats. The mechanism of this effect was attributable to HO-1 activation, as evidenced by andrographolide-induced HO-1 expression in brain tissues, which was highly localized in the cerebral capillary. HO-1 plays a vital role in neuroproteion as it degrades free heme and its metabolites. Together, it is established that andrographolide provides protection against MCAO-induced brain ischemic injury and neurological deficits via increased Nrf2-HO-1 expression through p38MAPK regulation (Figure 1).

### 1.2. Inhibition of VSMC and CEC Dysfunction Represents Neuroprotection of Andrographolide

Aberrant vascular smooth muscle cell (VSMC) proliferation induced by vascular injury has been shown to play a critical role in the pathogenesis of cardiovascular diseases (CVD) [13]. Therefore, suppression of VSMC and CEC cellular events would be candidate agents for treating CVDs. Chen et al. investigated the conceivable mechanisms of the inhibitory effects of andrographolide in VSMCs exposed to a proinflammatory stimulus, tumor necrosis factor-α (TNF-α) [14]. TNF-α-stimulated VSMCs treated with andrographolide suppressed the expression of inducible nitric oxide synthase (iNOS). A reduction in TNF-α-induced JNK, AKT, and p65 phosphorylation was detected in andrographolide-treated VSMCs [14]. However, andrographolide affected neither IκBα degradation nor p38MAPK or ERK1/2 phosphorylation in TNF-α-stimulated VSMCs. Treatment with both LY294002, a phosphatidylinositol 3-kinase/AKT inhibitor, and treatment with SP600125, a JNK inhibitor, markedly reversed the andrographolide-mediated inhibition of p65 phosphorylation. In addition, LY294002 and SP600125 both diminished AKT phosphorylation, whereas LY294002 had no effects on JNK phosphorylation. These results collectively suggest that therapeutic interventions using andrographolide can benefit the treatment of vascular inflammatory diseases, and andrographolide-mediated inhibition of NFκB activity in TNF-α-stimulated VSMCs occurs through the JNK-AKT-p65 signaling cascade [15]. This study also found that andrographolide induced p38MAPK could lead to activate p53 phosphorylation and the phosphorylated p53 consequently transactivated the expression of Bax. Andrographolide also activated the Src homology 1 domain-containing protein tyrosine phosphatase (SHP-1), and induced protein phosphatase 2 A (PP2A) dephosphorylation, both of these events were found to be inhibited by the SHP-1 inhibitor sodium stibogluconate (SSG) or shp-1 siRNA. SSG or shp-1 siRNA prevented andrographolide-induced apoptosis. These findings proposed that andrographolide triggers PP2A-p38MAPK-p53-Bax cascade, recovered mitochondrial dysfunction and VSMC death through an SHP-1-dependent mechanism [14].

**Figure 2:**
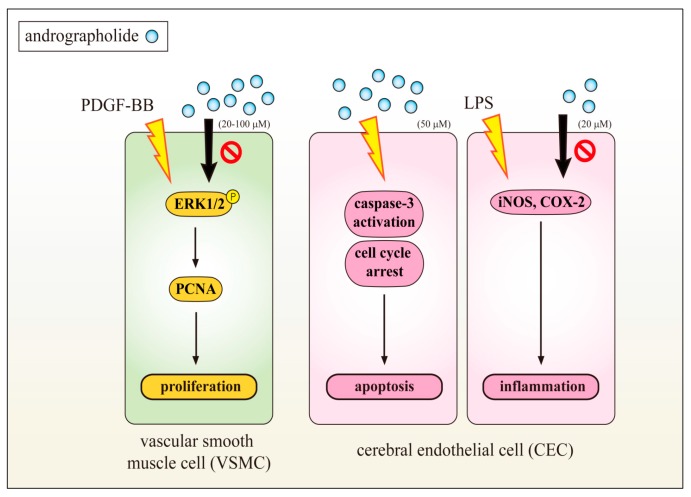
Andrographolide inhibited platelet-derived growth factor-BB (PDGF-BB) induced vascular smooth muscle cell (VSMC) proliferation via reducing extracellular signal-regulated kinase (ERK1/2), and inhibiting the expression of proliferating cell nuclear antigen (PCNA) in the G1 and S phases of the cell cycle. Andrographolide also reduced lipopolysaccharide (LPS)-induced inducible nitric oxide synthase (iNOS) and cyclooxygenase-2 (COX2) expression in inflammatory cerebral endothelial cells (CECs). Andrographolide induced CEC apoptosis and caspase-3 activation, and also arrested CEC cell cycle at the G0/G1 phase. These results suggested that the effects of andrographolide against VSMCs proliferation and CECs dysfunction may represent a promising approach for treatment of vascular diseases.

It is well-established that different stimuli are involved in the pathophysiology of cardiovascular diseases, which stimulate ERK1/2, p38MAPK and JNK in vascular cells [16]. ERK1/2 activation considered being required for its mitogenic signaling through a number of tyrosine kinase growth factor receptors, and the upregulation of platelet-derived growth factor receptor (PDGF-R) expression is known to be associated with the development and progression of proliferative cardiovascular diseases such as hypertension and atherosclerosis [17,18]. Therefore, an understanding of inhibition on PDGF-BB stimulated VSMC proliferation is important in terms of evolving approaches of treating cardiovascular disease. With the supporting of this hypothesis, an earlier study found that andrographolide expressively inhibited PDGF-BB induced VSMC cell proliferation via reducing ERK1/2, and inhibiting the expression of proliferating cell nuclear antigen (PCNA), a protein synthesized early in the G1 and S phases of the cell cycle, roles in cell progression, DNA replication and its repair (Figure 2). PCNA is a very sensitive indicator of measuring cell proliferation including VSMC proliferation [19]. Andrographolide also remarkably reduced lipopolysaccharide (LPS)-induced iNOS and cyclooxygenase-2 (COX2) expression [19]. The results of this study suggested that the effects of andrographolide against VSMCs proliferation and CECs dysfunction may represent a promising approach for treatment of vascular diseases. The data also revealed that andrographolide induced CEC apoptosis and caspase-3 activation, and also arrested CEC cell cycle at the G0/G1 phase (Figure 2). In addition, andrographolide at 5 mg/kg caused deterioration of MCAO/reperfusion-induced brain injury in a rat model [20]. Chen et al. characterized the apoptosis-inducing activity and mechanisms of andrographolide in rat VSMCs [21], and they found that andrographolide significantly induced ROS formation, p53 activation, Bax, and active caspase-3 expression, and these phenomena suppressed in N-acetyl-L-cysteine, a ROS scavenger, or diphenylene iodonium, a nicotinamide adenine dinucleotide phosphate (NADPH) oxidase (Nox) inhibitor pretreated VSMCs. In addition, p47phox, a Nox subunit protein was found to be phosphorylated in andrographolide-treated rat VSMCs. However, pretreatment with 3-O-methyl-sphingomyelin, a neutral sphingomyelinase inhibitor significantly inhibited andrographolide-induced p47phox phosphorylation as well as Bax and active caspase-3 expression. Altogether, this demonstrates that reducing the cell viability of andrographolide can be attributed to apoptosis in VSMCs, and this activity can be associated with the ceramide-p47phox-ROS signaling cascade [21]. These effects may provide strong information that andrographolide could be a therapeutic agent for treating ischemic stroke or neurodegenerative diseases.

### 1.3. Antiplatelet Action of Andrographolide Conferring Neuroprotection

**Figure 3:**
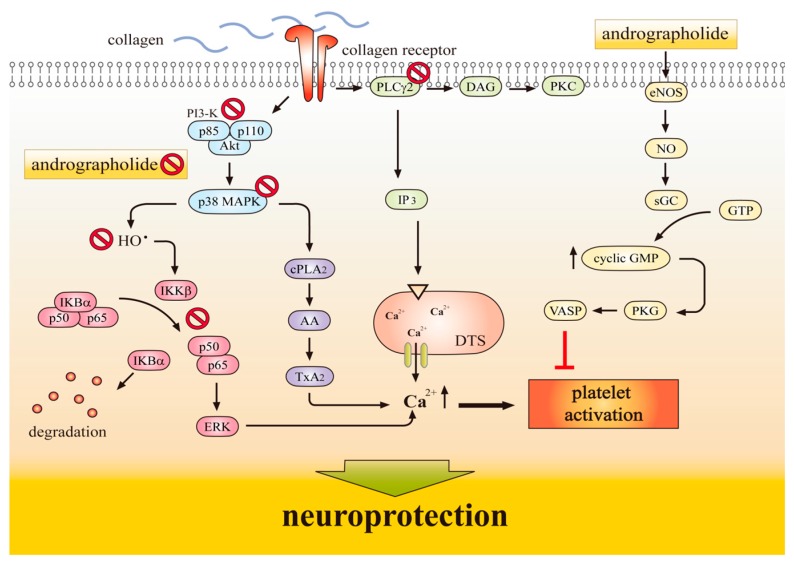
Andrographolide’s antiplatelet effects conferring neuroprotection. Collagen binds to its receptors and then activates both the PLCγ2-DAG-PKC and PI3 kinase/AKT-p38MAPK cascades. p38 MAPK can activate cPLA2, which catalyzes AA release to produce TxA2 formation. Andrographolide can activate the eNOS–NO–cyclic GMP pathway, followed by the inhibition of both the PLCγ2-DAG-PKC and PI3 kinase/AKT cascades, and ultimately inhibits platelet aggregation. Collagen triggers p38MAPK activation and hydroxyl radical (OH^−^) formation, followed by activation of NFκB including IKKβ phosphorylation, IκBα protein degradation, and p65 phosphorylation, subsequent activation of ERK2 phosphorylation, and finally triggering of [Ca^2+^]i mobilization and platelet activation. Andrographolide activates cyclic GMP/cyclic GMP-dependent kinase (PKG), and then inhibits the p38 MAPK-HO^−^-NFκB-ERK2 cascade which finally inhibits platelet activation. These findings suggested that the effects of andrographolide on platelet function may contribute to its neuroprotective effects. sGC: soluble guanylate cyclase; VASP: vasodilator-stimulated phosphoprotein; DTS: dense tubular system; AA: arachidonic acid; TxA2: thromboxane A2.

An intravascular thrombosis is among the generators of a wide variety of cardiovascular diseases. Introduction of an intraluminal thrombosis is supposed to involve platelet adherence and aggregation. Thus, platelet aggregation may play a crucial role in the atherothrombotic process [22]. Platelet activation and aggregation are common denominators in arterial thrombosis and various inflammatory diseases. This anuclear cell has been regarded absolutely as mediator of thrombosis and hemostasis; it function has been prolonged to include noticeable roles in inflammation and immunity [23]. Hence, the use of antiplatelet agents, which can inhibit thromboembolic diseases such as myocardial infarction, ischemic stroke, and vascular death in the platelets are warrant investigations. Amroyan et al. [24] found andrographolide inhibited platelet activating factor (PAF)-induced human platelet aggregation. Thisoda et al. [25] found that the extract of *A. paniculata* at 10–100 µg/mL significantly inhibited platelet aggregation in washed rat platelets. A previous study demonstrated that andrographolide exhibits potent antiplatelet activity through the activation of the endothelial nitric oxide synthase (eNOS)-NO/cyclic GMP pathway and inhibition of both the phospholipase Cγ (PLCγ)-protein kinase C (PKC) and phosphoinositide 3-kinase (PI3) kinase/AKT MAPK cascades in washed human platelets [26]. This study also showed that andrographolide may involve an increase in cyclic GMP/PKG, followed by inhibition of the p38MAPK/hydroxyl radicals (OH^−^)-NF-κB-ERK2 cascade in activated platelets (Figure 3).

A comprehensive in vitro study found andrographolide markedly inhibited collagen-stimulated platelet activation through modulating Ca^2+^ mobilization, thromboxane A(2) formation, and PLC-γ2, PKC, MAPK, and AKT phosphorylation. Andrographolide evidently increased cyclic GMP, but not cyclic AMP levels, and this compound also stimulated eNOS expression, NO release, and vasodilator-stimulated phosphoprotein (VASP) phosphorylation. Lu et al. [27] also found andrographolide reduced collagen-triggered OH^–^ formation. In vivo studies revealed that andrographolide (22 and 55 μg/kg) is effective in reducing the mortality of ADP-induced acute pulmonary thromboembolism and significantly prolonged platelet plug formation in mice. This study demonstrates that andrographolide possesses a novel role of antiplatelet activity via activation of the eNOS-NO/cyclic GMP pathway, resulting in the inhibition of the PI3 kinase/AKT-p38 MAPK and PLCγ2-PKC cascades, thereby leading to inhibition of platelet activation [27]. Together, it is suggested that andrographolide may have a high therapeutic potential to treat thromboembolic disorders.

Thisoda et al. [25] reported that andrographolide remarkably decreases thrombin induced platelet aggregation in a concentration-and time-dependent manner. They demonstrated that the down-regulation of ERK2 phosphorylation plays, in part, a role in the antiplatelet aggregation of andrographolide in thrombin-induced rat platelet aggregation. Wang et al. [28] found that andrographolide abolished the deposition of leucocytes (mainly CD68+macrophages) in the injured arterial walls by reducing the up-regulation of NF-κB target genes, including tissue factor, E-selectin and vascular cell adhesion molecule 1 (VCAM-1). In addition, this compound was protective against deep vein thrombosis in a murine model [29]. A study also found that apoptotic signaling events of caspase-3, -8, and Bid was time (10–60 min)-and dose (25–100 μΜ)-dependently activated by andrographolide in human platelets. Bid, a proapoptotic molecule of the Bcl-2 family and promoter of the release of cytochrome c is expressed in the brain, activated by cerebral ischemia in vivo, and contributes to ischemic cell death. A study found Bid in the cytosol of mouse brain and of primary cultured mouse neurons and showed that neuronal Bid is a substrate for caspase 8 [30]. This proapoptotic molecule found to be cleaved in vivo 4 h after transitory occlusion of the middle cerebral artery. Moreover, Bid (^−/−^) mice reported to a significant attenuation of infarction and significantly lower release of cytochrome c [30]. These findings indicate that the proapoptotic molecule Bid may contribute to the demise of nerve cells from cerebral ischemia by release of cytochrome c and activation of caspase. Moreover, z-IETD-fmk, a caspase-8 inhibitor was found to reverse andrographolide-induced caspase-8 activation, whereas the antagonistic anti-Fas receptor (ZB4) and anti-TNF-receptor (H398) monoclonal antibodies did not effective on this phenomenon [31]. Overall, all of these findings suggested that the effects of andrographolide on platelet function may contribute to its neuroprotective or antithrombotic effects (Figure 3).

## 2. Molecular Mechanisms Underlying the Neuroprotective Effects of Andrographolide

Currently, pharmaceutical therapies confer the major strategies for neuroprotective functions. Mechanism of action underlying the neuroprotection of pharmaceutical or natural compounds is by their interactions with signaling-related transporters, receptors, or key enzymes.

### 2.1. Neuroprotective Role of Andrographolide via HO-1 Induction

Hemoxygenase HO-1 is an inducible isoform of the first and rate-controlling enzyme of the degradation of heme into iron, carbon monoxide, and biliverdin, the latter being subsequently converted into bilirubin. Because of the numerous beneficial biological effects of HO-1, it has gained attention, as anti-inflammatory, antiapoptotic, angiogenic, and cytoprotective functions. Thus, the physiological stimulation of HO-1 may be a beneficial response to several noxious stimuli, including heme itself, suggesting a potentially autoprotective and autodefensive role in several pathophysiological states including stroke.

HO-1 expression is controlled by numerous signaling pathways and transcription factors, the most salient of which are Nrf2, activator protein (AP-1), and NF-κB [32]. Among them, the redox-dependent Nrf2 system plays a central role in HO-1 induction [33]. HO-1 has been identified to protect tissues by repairing redox homeostasis and tumbling inflammation because of its antioxidant, antiapoptotic, and antiinflammatory properties. HO-1 plays a vital role in neuroprotection because it degrades free heme and its metabolites, CO, biliverdin, or bilirubin, and these may directly provide cytoprotection. This enzyme has been considered particularly valuable in neuroprotection against cerebral ischemia, because HO-1 knockout mice reported to exhibit greater ischemic damage than did wild-type mice [34]. Ding et al. [35] reported that 11-keto-β-boswellic acid is a triterpenoid compound from Boswellia serrata extracts provides neuroprotection by inducing HO-1 expression. In another study, it has demonstrated that panaxatriol saponins, the main components of Panax notoginseng could effectively reduce oxygen-glucose deprivation (OGD), a widely used in vitro model of stroke- or reperfusion-induced injury by inducing HO-1 stimulation [36]. Resveratrol, a naturally occurring polyphenolic phytoalexin, is predominantly found in dietary sources, including grapes, red wine, peanuts, plums and other plants is reported to diminish injury and promoted proliferation of the neural stem cells by upregulating the expression of HO-1 following OGD/R injury in vitro [37]. A recent study also demonstrated that nicotinamide mononucleotide (NMN), a key intermediate of nicotinamide adenine dinucleotide (NAD^+^) biosynthesis protected against collagenase-induced intracerebral hemorrhage (ICH) in mice via inducing HO-1 expression [38]. Consistent with these findings, a study also exhibited that HO-1 knockdown partially reduced andrographolide-mediated protection while CECs were subjected to oxygen-glucose deprivation [39]. These observations provide compiling information that HO-1 activation contributes to the neuroprotection of andrographolide (Figure 1).

### 2.2. Andrographolide’s on Stroke Treatment via Inhibiting MAPK Signalling

Mitogen-activated protein kinases (MAPKs) are a group of serine/threonine protein kinases consisting of several members including ERK1/2, JNK and p38 [40]. MAPKs have central roles in signal transduction from the cell surface to the nucleus and control cell death and survival in both physiological and pathological conditions [41]. The importance of MAKPs in stroke has been well documented in the literature. More precisely, JNK activation has been shown to increase stroke injury via activation of neuronal apoptosis and both genetic and pharmacological inhibition of JNK improved outcomes after stroke [42,43]. P38 signaling activation impairs stroke-induced inflammatory responses and also leads to worse stroke outcomes [44]. A number of researchers have examined the important role of MAPK in the pathologic course of stroke. MAPK activation is also known to play physiological and pathological roles post-development, and there is a large body of evidence to suggest that MAPK also contributes to regulating inflammatory responses, cytokines, cell apoptosis, and death in ischemic and hemorrhagic brain injury [45]. Thus, inhibiting or regulating the expression and activity of MAPK signaling may constitute novel therapeutic strategies for stroke.

Several investigators have studied the expression, activity, and distribution of MAPK in ischemic stroke. The results have suggested a role for the MAPK pathway in the regulation of cytokine expression and cell apoptosis following experimental cerebral ischemia [45,46,47]. Several investigators have demonstrated that cerebral ischemia results in the time-dependent activation of ERK and JNK and that upstream MEK inhibition results in significant decreases in inflammatory cytokines and cell apoptosis in the injury area [45,46]. Maddahi and Edvinsson [48] evaluated the role of Raf-MEK/ERK1/2 signaling transducers in regulating the expression of pro-inflammatory mediators in cerebral vessels following ischemic stroke. Chang et al. demonstrated that platelet-derived growth factor-BB (PDGF-BB), the most potent proliferative factor, induces activation of ERK1/2 and this activation was significantly suppressed by andrographolide in a concentration-dependent manner [19]. A study from Chen et al. found that andrographolide activated the p38MAPK, leading to p53 phosphorylation, and phosphorylated p53 subsequently transactivated the expression of Bax, a pro-apoptotic protein [14]. Transfection with PP2A small interfering RNA (siRNA) suppressed andrographolide-induced p38MAPK activation, p53 phosphorylation, and caspase-3 activation. Andrographolide also activated the Src homology 1 domain-containing protein tyrosine phosphatase (SHP-1), and induced PP2A dephosphorylation, both of which were inhibited by the SHP-1 inhibitor sodium stibogluconate (SSG) or shp-1 siRNA. SSG or shp-1 siRNA prevented andrographolide-induced apoptosis. These results suggest that andrographolide activates the PP2A-p38MAPK-p53-Bax cascade, causing mitochondrial dysfunction and VSMC death through an SHP-1-dependent mechanism. This effect may provide an encouraging approach for the development of novel drug against vascular diseases.

### 2.3. Andrographolide’s Neuroprotection via Nrf2 Activation

The nuclear erythroid 2-related-factor 2 (Nrf2) transcription factor is a vital regulator of an essential neuroprotective reaction by pouring the expression of numerous cytoprotective genes through its interaction with the antioxidant response element (ARE). Dysregulation of the Nrf2-ARE system has been identified in human disease, especially Nrf2-ARE pathway considered as an attractive therapeutic target for neuroprotection. A previous study confirmed that both Nrf2 and multiple Nrf2 target genes exhibited reduced expression in a motor neuronal cell line expressing mutant (G93A) human superoxide dismutase (SOD1) [49]. A reduction of Nrf2 transcripts and protein in spinal motor neurons and motor cortex from cases of sporadic amyotrophic lateral sclerosis (ALS) has also been described [50]. Astrocyte-specific expression of Nrf2 delayed the onset and extended survival in two mouse models of ALS [51]. This pathway thus represents an attractive therapeutic target due to its disease-specific dysregulation and the convincing evidence for a role of oxidative stress in disease progression. In addition, it is a well-defined target, amenable to activation by small molecules and activation of intrinsic cellular defense mechanisms may confer a more effective and enduring protection against oxidative stress than, for example, direct free radical scavenging. A number of molecules have been described that activate the Nrf2-ARE pathway. A previous study reported that 11-keto-β-boswellic acid markedly increased Nrf2 expression in primarily cultured astrocytes, the major glial nonneuronal cells, and plays a critical role in cellular antioxidant defense in the brain [35]. Similarly, ursolic acid, a naturally occurring pentacyclic triterpenoid, was reported to promote neuroprotection after cerebral ischemia in mice by activating the Nrf2 pathway [52].

A previous study was compared the effectiveness of two synthetic Nrf2 activators, tert-butylhydroquinone and 2-cyano-3,12-dioxooleana-1,9-diene-28-imidazolide and 54 natural compounds on Keap1-Nrf2 pathway against detoxifying oxidative stress using AREc32 cell line, and the results found that among the tested natural compounds, andrographolide had the highest efficacy on inducing Nrf2 [53], which indicated that andrographolide may serve as candidates to protect against oxidative stress-induced pathogenesis via inducing Nrf2. A recent study found that phosphorylated Nrf2 expression increased time-dependently in andrographolide treated CEC [12], also found Nrf2 siRNA markedly downregulated andrographolide-induced Nrf2 and HO-1 protein expression. These results demonstrated that andrographolide induces HO-1 expression by activating the Nrf2 signaling pathway. In addition, this study was determined the role of p38MAPK in downregulating Nrf2 phosphorylation in CECs after andrographolide treatment. The data showed that SB203580, a p38MAPK inhibitor, significantly inhibited Nrf2 phosphorylation after 30 min of andrographolide treatment, suggested that p38MAPK may function as an upstream regulator of Nrf2 activation. Taken together, these results suggested that andrographolide enhanced HO-1 expression possibly through the p38MAPK-Nrf2 pathway in primary cultured CECs (Figure 1). Immunocytochemical staining study confirmed the increased nuclear localization of Nrf2 in CECs treated with andrographolide. Overall, it can be suggested that the andrographolide induced Nrf2/ARE pathway represents a promising drug target to the pathology of multiple neurodegenerative diseases.

### 2.4. NF-κB Inhibition Contributes Neuroprotection of Andrographolide

Nuclear factor kappa B (NF-κB) transcription factor also plays an important role in the inflammatory response by regulating the expression of various genes encoding pro-inflammatory mediators. In normal cells, NF-κB is present in the cytoplasm as an inactive heterodimer composed of two subunits, p50 and p65. The heterodimer is complexed with an inhibitory protein IκB-α. When cells activated by certain inflammatory agents, the IκB-α protein gets phosphorylated, causing its rapid degradation and, NF-κB becomes dissociated from IκB-α. Phosphorylated IκB-α is then rapidly degraded by the proteosome, leading to the translocation of NF-κB to the nucleus, where it binds to the specific DNA sequence present in the promoters of numerous target genes. Various natural compounds have been shown to exhibit anti-inflammatory activity through inactivation of NF-κB via different mechanisms [54]. A recent study demonstrated that coumaroyl lupendioic acid, a new compound, isolated from the stem bark of Careya arborea, inhibits inflammation in carrageenan-induced paw edema through the suppression of NF-κB signaling pathway [55]. Several lines of evidence indicate that inhibition of NF-κB contributes to the protective anti-inflammatory actions of andrographolide [56,57]. Andrographolide inhibits NF-κB activation by blocking the binding of NF-κB oligonucleotides to nuclear proteins [56]. Recently, we demonstrated that andrographolide enhances the NF-κB subunit p65 Ser536 dephosphorylation through the activation of PP2A in VSMC [52]. We also established that andrographolide inhibited p65 Ser536 phosphorylation, reduced nuclear translocation of p65, and diminished p65 kB oligonucleotide binding in LPS/interferon-γ (IFN-γ)-stimulated rat VSMCs [57].

NF-κB plays a pivotal role in the pathogenesis of inflammation, prompting various drugs designed to treat human inflammatory disease to be focused on inhibiting NF-κB activation [58]. Many natural compounds or herbal extracts reportedly exhibit anti-inflammatory activities that generally involve NF-κB activation [59,60]. Phytochemicals are currently of interest due to their essential biological and pharmacological properties, including the inhibition of NF-κB activation [61]. The NF-κB transcription factor regulates the expression of various components of the immune system, including proinflammatory cytokines, chemokines, adhesion molecules, and inducible enzymes such as COX2 and iNOS, as well as proteins that regulate the specific immune response, such as interleukin-(IL-)2, IL-12, and interferon-γ that control lymphocyte proliferation and differentiation. Therefore, dysregulation of this transcription factor can lead to inflammatory and autoimmune diseases [62]. Andrographolide has been proven to attenuate inflammation by inhibiting NF-κB activation through the covalent modification of reduced Cys62 of p50. Systematically, andrographolide formed a covalent adduct with a reduced cysteine of p50, thus blocking the binding of NF-κB oligonucleotide to nuclear proteins.

A reduction in TNF-α-induced JNK, AKT, and p65 phosphorylation was observed in andrographolide-treated VSMCs. However, andrographolide affected neither IκBα degradation nor p38MAPK or ERK1/2 phosphorylation TNF-α-induced VSMCs. Treatment with LY294002, a phosphatidylinositol 3-kinase/AKT inhibitor, and treatment with SP600125, a JNK inhibitor, markedly reversed the andrographolide-mediated inhibition of p65 phosphorylation. In addition, LY294002 and SP600125 diminished AKT phosphorylation, whereas LY294002 had no effects on JNK phosphorylation. A study from Chan et al. [10] demonstrated that andrographolide produced neuroprotective effects against cerebral ischaemia with associated inhibition of microglia activation, possibly caused by the suppression of NF-κB activation, leading to a reduction in the production of cytokines including TNF-a and IL-1b, and pro-inflammatory factors such as PGE2. Together, studies suggest that therapeutic interventions using andrographolide can benefit the treatment of vascular inflammatory diseases, and andrographolide-mediated inhibition of NF-κB activity in TNF-α-stimulated VSMCs occurs through the JNK-AKT-p65 signaling cascade, an IκBα-independent mechanism [15].

### 2.5. Andrographolide Improves Neuronal Functions via Induction of Apoptosis

The brain endothelium constitutes a barrier to the passive movement of substances from the blood into the cerebral microenvironment [63]. In addition to regulating vasomotor tone, platelet adhesion and neovascularization, CECs play a crucial role in supporting the integrity and the function of the BBB [60]. However, CEC apoptosis was suggested to disrupt the BBB integrity, subsequently leading to brain edema and neuronal damage [64]. Cheng et al. [65] demonstrated that ischemia-induced apoptosis of CECs was mediated by increased expression of p53 and activation of caspase-3 signaling. Deposition of b-amyloid (Ab) also induced CEC death via caspase-8/ROS/apoptosis signal-regulating kinase 1 (ASK) 1-dependent apoptosis [66]. So, protection of the BBB from apoptosis may be a beneficial therapeutic strategy for neuronal functions. Previous study found that treatment with andrographolide at 50 and 100 mM for 24 h induced CEC apoptosis. Andrographolide also time-dependently triggered caspase-3 activation, indicating that andrographolide induces cell apoptosis, ultimately leading to cell death or cell necrosis in the late stage [20]. (In addition, this compound markedly increased the cell distribution in the G0/G1 phase, which advocates that andrographolide may arrest cell growth in the G0/G1 phase and hamper cell entry into the S or G2/M phase, ultimately inhibiting CEC growth [20]. This study also found that andrographolide causes decline of MCAO/reperfusion-induced brain injury, as it proposes that the diminishing effect of andrographolide on brain injury, at least in part, is associated with its ability to persuade CEC apoptosis [20]. Moreover, earlier studies displayed that andrographolide at a relatively low dose (0.1 mg/kg) could protect neuronal function against ischemia/reperfusion-induced brain injury in a rat or mouse model [10,67]. This corroboration indicates that andrographolide at a rather low dose (0.1 mg/kg) protects against brain injury, but it aggravates brain injury at a higher dose (5 mg/kg).

## 3. Conclusions

Advancement of protective agents from natural products is an auspicious direction in the treatment of ischemic cerebral injury and related neurodegenerative diseases. Thus, more attention has been paid to natural compounds as they can drop the BBB, extend therapeutic time windows, clear pharmacological targets and have fewer the side effects. Andrographolide has antioxidant and anti-inflammatory bioactivities. In vitro and in vivo studies showed that it exerts neuroprotection at low doses. This review provides a molecular mechanism for andrographolide amendment of brain injury in ischemic stroke. As a potent anti-inflammatory drug with neuroprotective activities, the favorable effects of andrographolide to treat ischemic stroke in humans warrant further basic and clinical investigations.
